# Whole-genome sequencing and phylogenetic analysis of rabies viruses from Jordan

**DOI:** 10.1371/journal.pntd.0009431

**Published:** 2021-05-20

**Authors:** Laith N. AL-Eitan, Guanghui Wu, Megan Golding, Yue Tang, Hooman Goharriz, Denise A. Marston, Anthony R. Fooks, Lorraine M. McElhinney

**Affiliations:** 1 Department of Biotechnology and Genetic Engineering, Jordan University of Science and Technology, Irbid, Jordan; 2 Animal and Plant Health Agency (APHA, Weybridge), Surrey, United Kingdom; University of Glasgow, UNITED KINGDOM

## Abstract

Human fatalities caused by rabies are rarely reported in Jordan; however, domestic animals are more likely to fall victim to rabies compared to wild animals, at least this is the case in Jordan due to the presence of canine rabies. In this study, twelve brain samples from domestic and wild animals suspected of being infected with rabies virus from different regions of Jordan were collected during 2019. Seven of them tested positive using the fluorescent antibody test and real-time SYBR RT-PCR assay. Five specimens were from stray dogs and two from foxes. The whole genome sequences were obtained from the positive samples. Sequence analysis showed that one dog virus from Al Quwaysimah city located in Amman governorate, was closely related to an Israeli strain belonging to a Cosmopolitan ME1a clade. The genomes of the remaining six viruses (four from dogs and two from foxes) collected from different areas of Jordan were genetically-related to each other and clustered together with sequences from Iran and Turkey; all belong to Cosmopolitan ME2 clade. These sequences were analyzed with six other Jordanian rabies virus nucleoprotein (N) gene sequences available in the public database, five of them belong to ME1a clade and one belongs to ME1b clade. Rabies virus whole genome data is scarce across the Middle East. This study provides a better understanding of the molecular epidemiology of rabies virus in the region.

## Author summary

In this study, we performed whole genome sequencing (WGS) for rabies virus (RABV) isolates from seven samples, five of which were of stray dogs, and the other two were from foxes. Specimens were collected from animals across Jordan, including Balqa, Amman, Irbid, Tafilah, and Madaba governorates. Six out of the seven isolates were belonging to the Cosmopolitan ME2 clade, which related to the Iranian and Turkish sequences. This is not the case previously, where the majority of the Jordanian isolates belong to Cosmopolitan ME1a clade and closely related to the sequences from Israel. This shift might be due to the applied regulations across borders between Jordan and Israel. Besides the growth in travel and trade movement between Jordan and Turkey, where the latter is a border country with Iran. These collected data, where such studies are not common in the Middle East countries, will enhance our understanding of the RABV evolution and epidemiology in the region for rapid and effective response for rabies virus outbreaks.

## Introduction

Annually, canine rabies death tolls estimated by 59,000 human deaths worldwide and over 3.7 million disability-adjusted life years (DALYs) [1]. Human rabies is a rare occurrence in Jordan, and the Ministry of Health (MOH) provides post-exposure prophylaxis for any individual afflicted by a stray or owned dog bite and is responsible for investigating rabies virus infection. [2,3]. In an epidemiological study of 419 brain samples from animals suspected of rabies collected by Jordan’s MOH, among the 164 positive animals, the majority were from stray dogs (45.1%) and cattle (19.5%) [4]. In 2017, a young girl was bitten by a rabid dog in the northern governorate of Irbid, and despite the fact that her family immediately sought medical help, she was sent home without being given post-exposure vaccination, which culminated in her death from rabies a few weeks later [5]. Due to the lack of national surveillance of rabies and insufficiency in animal screening, prevalence in animals is not fully understood or reported, which is a major health issue.

A recent study reported that the primary diagnostic test for rabies in Jordan, the fluorescent antibody test (FAT), was not conclusive when used on its own, as it resulted in false positive results for some samples [6]. Samples reported by the testers as FAT positive, were subsequently found to be negative by histopathology and RT-PCR, and therefore, it was recommended to include other tests in addition to the FAT [6]. In addition, the FAT must be undertaken on the appropriate brain samples, by highly skilled staff using validated reagents. Furthermore, there still appears to be a substantial amount of misinformation and lack of awareness of rabies both in the community and among medical professionals. It is imperative to both enhance the diagnostic capability and educate the general public and medical professionals in Jordan.

Rabies virus (RABV) is a member of the *Lyssavirus* genus and is responsible for a much-feared zoonosis that is almost always fatal once symptoms become apparent in infected individuals [7]. Classical symptoms of rabies are generally non-specific initially, include fever, headache, and fatigue but eventually progress to acute neurological symptoms of encephalitis, paralysis, delirium to coma, and ultimately death. Meanwhile, non-neurologic signs are developed and affect cardiac, respiratory, and gastrointestinal organs, where respiratory failure is the main cause of patients’ death [8–13].

Lyssaviruses are a group of neurotropic single-stranded RNA viruses with a genome of approximately 12 kb that encodes five structural proteins (nucleoprotein (N), phosphoprotein (P), matrix protein (M), glycoprotein (G), and RNA dependent RNA polymerase (L) [14]. The intragenic regions that separate these genes are varied in length. Phylogenetic classification of rabies virus variants is often based on partial gene sequences indicative of specific genes [14]. However, when whole genome sequences are used for the phylogenetic classification, it allows for a more complete study on the evolution, spread, and genome-wide heterogeneity of viruses [15]. However, there is limited complete rabies virus genome sequence data and phylogenetic analysis undertaken in the Middle East region [3,16–18]. There are a number of phylogenetic analyses using partial nucleoprotein (N) gene sequence data, which revealed the presence of several phylogenetic lineages of rabies virus in the Middle East [3,19]. In this study, whole genome characterization of rabies viruses circulating in Jordan was undertaken.

## Materials and methods

### Materials

Twelve brain tissue samples were obtained from various regions of Jordan from animals suspected of rabies (S1 Table).

### Rabies diagnostic tests

The twelve samples tested positive in Jordan using the fluorescent antibody test (FAT) and were re-tested at the OIE Reference Laboratory for rabies using OIE terrestrial manual [20], at the Animal and Plant Health Agency (APHA), by FAT and by SYBR real-time RT-PCR method [21]. Tissue samples were treated with OmniCleave Endonuclease (Epicentre, Illumina, US) to deplete host DNA then extracted by TRlzol LS (ThermoFisher Scientific, USA) and RNeasy Mini Kit (Cat No./ID: 74004) following manufacturer’s instructions. For the Real-time PCR Sybr green, the RNA samples were amplified using the highly sensitive iTaq Universal SYBR Green One-Step RT-PCR Kit. Following the manufacturer’s instructions, using the N165-146 and JW12 RT PCR primers provided a final master mix is prepared in a single tube per sample. Using Mx3000P real time PCR machine (Stratagene), the PCR thermal profile consists of; 1 cycle at 50°C for 10:00 minutes for cDNA synthesis, 1 cycle at 95°C for 5:00 min for reverse transcriptase inactivation, 40 cycles at 95°C for 00:10 seconds, then, 60°C for 00:30 seconds respectively for PCR cycling and detection. Finally, Melt curve analysis, 1 cycle, 95°C for 1:00 min, 55°C for 1:00 min, then 95°C for 00:10 sec.

### Whole genome sequencing

Extracted RNA was submitted to APHA Central Sequencing Unit (CSU) for next generation sequencing (NGS). Briefly, superscript II reverse transcriptase was used to obtain the viral cDNA. The cDNA concentration was normalized and delivered to CSU in a 96 well plate with corresponding NGS submission form. Using Omega MARS software to achieve a standard concentration of 0.2 ng. The sequencing library was prepared using a NexteraXT library kit (Illumina, Cambridge, UK) and sequencing libraries were run on an Illumina NextSeq sequencer.

### Assembly of genomic sequences

The raw read data was mapped to reference sequence, accession no. KY860612, using the Burrow-Wheeler Aligner (version 0.7.17-r1188) [22]. A consensus sequence was then generated using SAMtools v1.10 [23] and vcf2consensus.pl script (available at: https://github.com/ellisrichardj/csu_scripts/blob/master/vcf2consensus.pl). This was repeated for three subsequent iterations of mapping, with each intermediate consensus sequence being used as the reference for the following mapping iteration. The final consensus sequences (4^th^ iteration) were visualised in Tablet v1.17.08.17, to inspect read alignment and genome coverage (Table 1). All seven genomes are 100% covered. The lowest one is LNB, only the first 60 nucleotides have a read depth are below 30. Sequencing resulted in 100% coverage of the five lyssavirus genes for all seven genomes. The whole genome sequences were submitted to the European Nucleotide Archive (ENA) database (accession no. PRJEB41551).

**Table 1:** The coverage summary values (read depth).

Sample	Mini	Max	Average
LN2	65	19149	9595.679
LN3	6	896	372.8989
LN5	2	865	277.4739
LN9	99	60920	23842.01
LN12	21	5218	2289.938
LNA	31	18583	7621.321
LNB	1	878	340.365

### Phylogenetic analysis

ClustalW (MegAlign, DNASTAR Version 15.0.0) was used for sequence alignment of whole genome and N gene sequences. Moreover, Snippy was used for phylogenetic analysis (https://github.com/tseemann/snippy) to produce a core genome pseudo FASTA file using KX1481892 (the sequence of an Israeli dog virus) as the reference. The pseudo FASTA file was input to Raxml [24] to produce a Newick file and to HierBAPS [25] to analyse population structure. The results were uploaded to Tree of life (iTol) to product midpoint rooted trees [26]. The default settings of the program were used for the analysis. RABV-GLUE assignment agreed with HierBaps assignment or was supported by phylogenetic clustering (http://rabv.glue.cvr.ac.uk/#/rabvFastaAnalysis).

## Results and discussion

The twelve brain samples that tested positive for rabies (FAT) in Jordan were re-analyzed in APHA, UK and seven were confirmed as positive for rabies virus (S1 Table and S1 Fig). Representative images of the FAT results are shown (see S2 Fig). The results were confirmed by the real-time RT-PCR SYBR green assay (results not shown) [27].

All seven confirmed positive tissues were from either foxes (*Vulpes vulpes*) or dogs *(Canis lupus familiaris*). Three samples collected from sheep (*Ovis aries*) and cows (*Bos taurus*) were taken immediately after they were bitten by stray dogs and before any clinical signs of rabies could have developed. These samples would not be expected to yield positive results by FAT. No samples from the associated stray dogs were obtained. It was therefore not evident whether the stray dogs were infected or not. Nevertheless, the threat of rabies in domesticated animals was highlighted in an earlier study [4]. The two dog samples that were discordant, may have been either false positives in the original diagnosis or may have deteriorated over time or during transit to the UK. The use of RT-PCR as an alternative or confirmatory test for rabies in quality assured laboratories is now accepted by the OIE. A previous study of rabies diagnosis in decomposed carcasses showed that the use of RT-PCR was recommended in addition to other conventional methodologies in routine rabies diagnosis, particularly for decayed samples [28].

**Fig 1:**
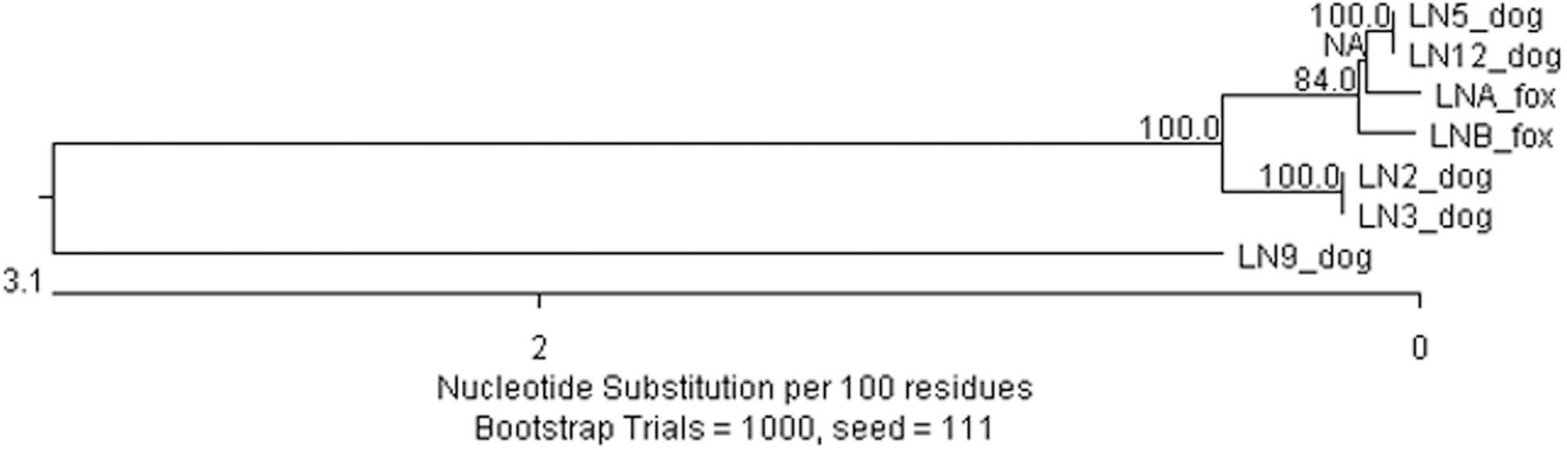
Phylogenetic tree generated by ClustalW (MegAlign, DNASTAR Version 15.0.0).

The whole genomes of seven positive samples were sequenced and assembled (see Figs 1 and S3). The genomes of six viruses clustered together, but one virus was significantly different. Interestingly, the six genetically related viruses were from different areas of Jordan. Viruses with nearly identical genome sequences (LN2/LN3 and LN5/LN12) were from distant geographical areas in Jordan (See S1 Fig), which suggests a wide dissemination of the same strain of rabies virus within Jordan. The whole genome sequence of one virus from AlQuwaysimah city (LN9_2019_Jordan_Dog) in Amman governorate, had >98.8% identity with a cattle virus from Israel.

**Fig 2:**
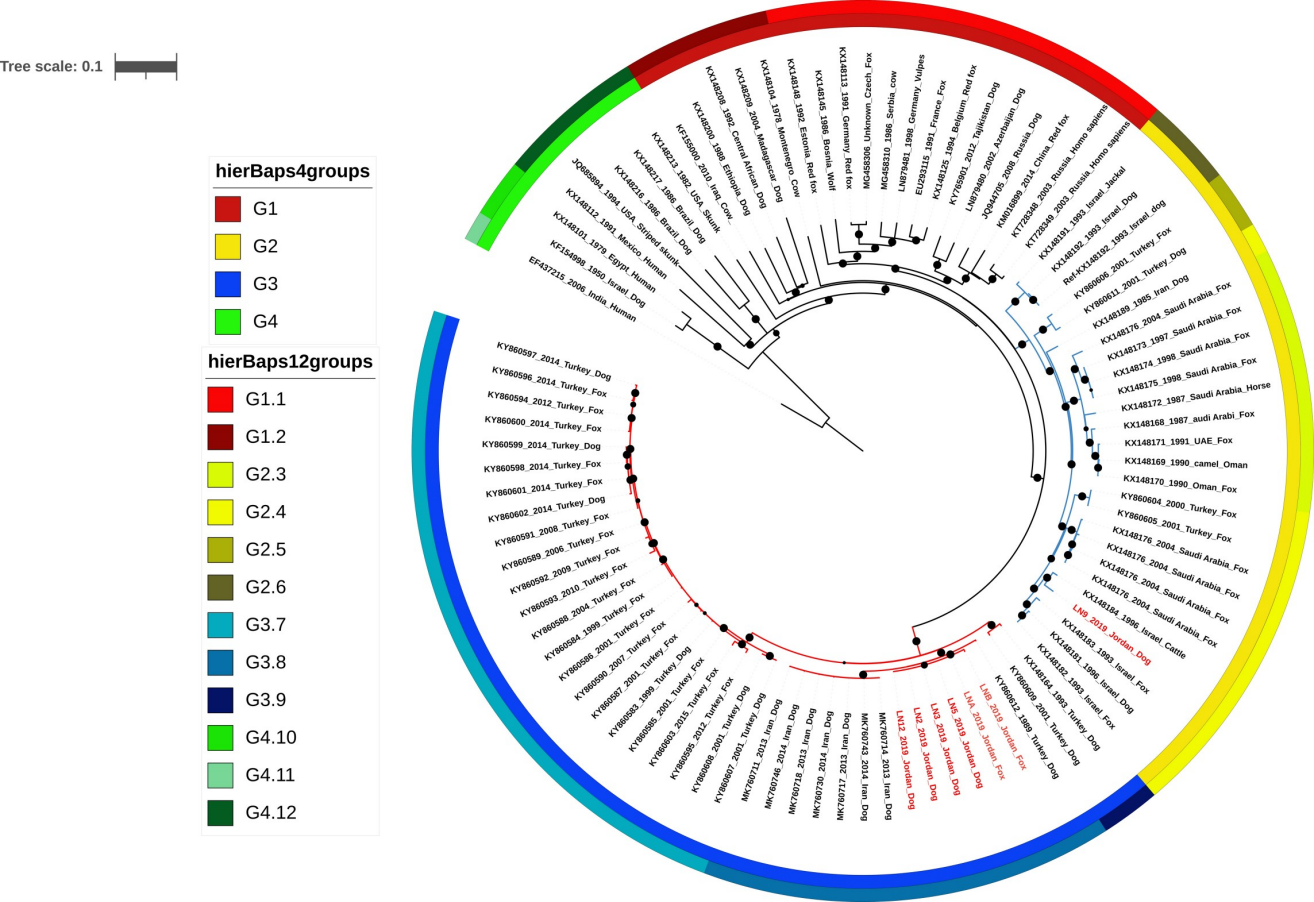
Phylogenetic tree shows the relationships between the Jordanian viruses (*n = 7*, FASTQ files) and nearby countries plus other representative viruses worldwide (*n = 85*, genome sequences). The analysis was performed using Snippy and population structure generated using HierBAPS (colored bars). All analysis was performed with the default setting of the program. The midpoint rooted tree is produced by iTol. KX148192, the sequence of an Israeli dog virus was used as the reference. The phylogenic clades of viruses are also indicated. Bootstrap results are included (black circles) and only those with bootstrap values >70% are shown, the largest circles are 100%. The ME1 clade sequences are in G2 (G2.3, G2.4, G2.5 and G2.6), and those of ME2 clade are in G3 (G3.7, 3.8 and 3.9).

Phylogenetic analysis was performed using Snippy to include 85 complete RABV genomes representing major clades of canine RABV and whole genome sequences from nearby countries (Fig 2). These 92 sequences (including seven sequences from this study) formed four major groups and twelve sub-groups. As fewer sequences were selected from distantly related sequences, multiple clades were grouped together. As all available sequences from nearby countries were included, the same clades were further divided into different groups to increase the resolution (Fig 2). The sequences belong to Cosmopolitan ME1 are in G2 and all Cosmopolitan ME2 sequences are in G3. G2 is subdivided into G2.3, G2.4, G2.5 and G2.6. The sequences in G2.4 and G2.5 corresponds to ME1a and those in G2.6 corresponds to ME1b defined by Troupin et al., 206 [19]. The sequences in G2.3 were not included in the analysis performed by Troupin [19]. As more sequences from the Middle East were included in our analysis, we have obtained higher resolution. The same is ME2, it can be subdivided into 3 groups based on sequences were have got so far G3.7, 3.8 and 3.9 (Fig 2). Agreeing with the early alignment (Fig 1), 6 of the seven sequences from Jordan grouped together all belonging to the Cosmopolitan ME2 clade and G3.8., neither countries share a border with Jordan. There is a lack of RABV whole genome sequences in the immediate neighbors of Jordan such as Saudi Arabia, Iraq, and Syria. In principle, viruses from those countries should have a higher similarity to Jordanian viruses. These are neighboring countries and there is a possibility of boarders’ cross dogs. One Jordanian dog rabies virus (LN9) belongs to a Cosmopolitan ME1a clade, which is most closely related to sequences of Israeli cattle, fox, and dog viruses as well as fox viruses from Turkey.

There are minor differences between the phylogeny of whole genome sequences (Fig 1) and phylogeny based on the core genome of 92 genomes (Fig 2 and S1 Appendix). There are some differences amongst whole genomes of four Jordanian dog viruses (LN, 2, 3, 5 and 12, Fig 1), whereas in SNP core genome analysis, all dog rabies viruses were identical but differ from fox viruses by 11 or 12 SNPs. Additionally, two fox viruses differ from each other by 23 SNPs (S1 Appendix). As the core genome was taken from the shared regions of 92 genomes; the highly variable regions were excluded from this analysis.

**Fig 3:**
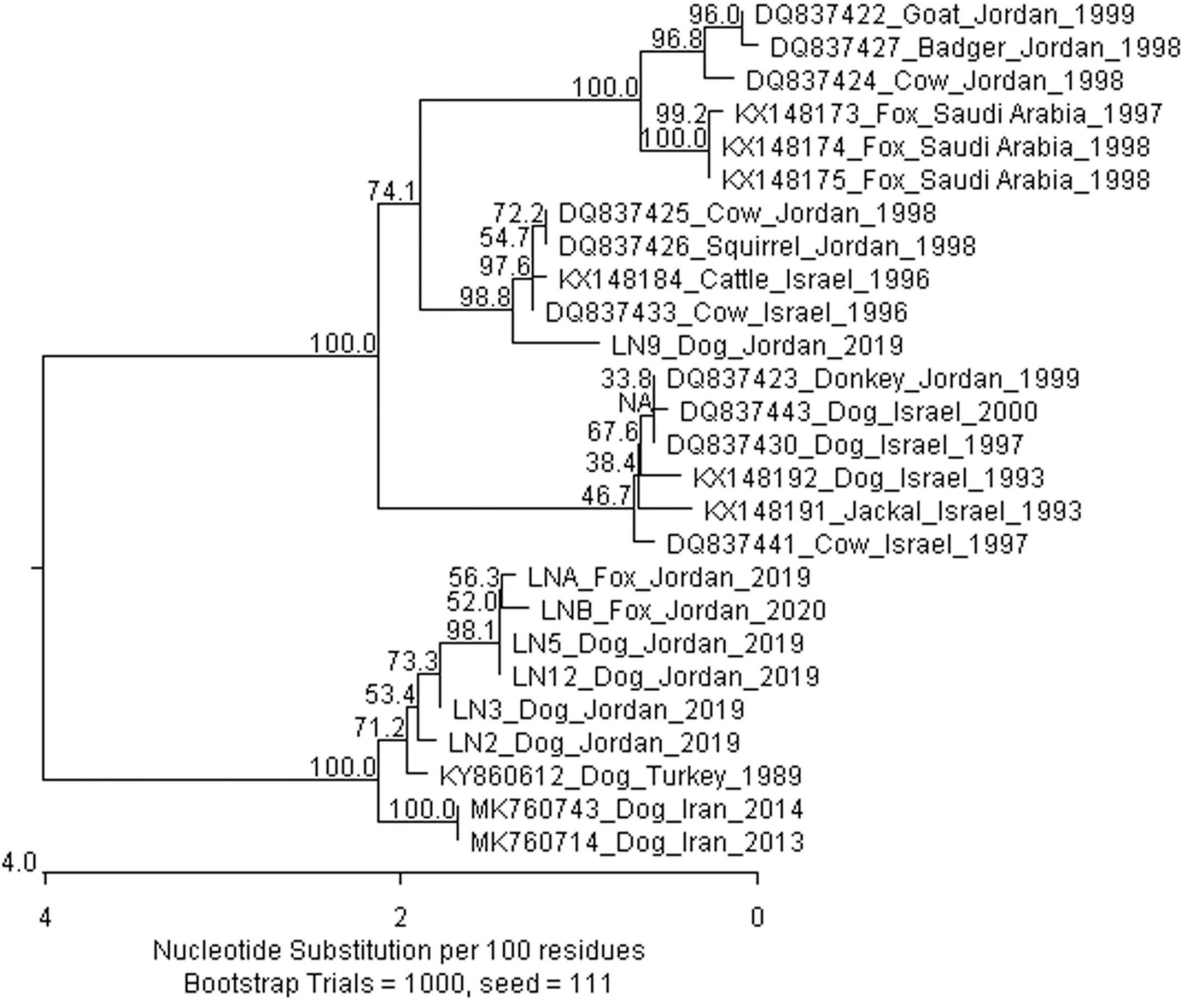
Phylogenetic tree generated by ClustalW (MegAlign, DNASTAR Version 15.0.0) using the sequences of the N genes from this study and those found from nearby countries.

Although whole genome sequences are lacking in Jordan, six rabies virus N gene sequences were found in the NCBI nucleotide database. They were analyzed together with the N gene sequences from this study and other closely related sequences from nearby countries (See Fig 3). Five of these historic sequences belonging to the ME1a clade were from various animals, but one sequence from a donkey (*Equus asinus*) belonged to ME1b clade similar to LN9. There is relevant information on the geographic locations of these earlier sequences in Jordan [3].

Although rabies is endemic in animals in the Arabian countries [2,4], there is minimal information with details of rabies virus molecular epidemiology in the Middle East, especially in Jordan [3].. More than 50% of human rabies cases in Jordan were in the northern region of the country (Haddadin et al., 2007). The majority of the reported cases were of dog bites, according to the MOH reports [2]. Although the Ministry of Agriculture (MOA) provides free vaccination for rabies, there is inadequacy in the vaccines, which limited the coverage of the nationwide population of animals, particularly the stray dog population [2]. The data obtained in this study makes an essential contribution to our understanding of RABV epidemiology and its relationship with host species in Jordan. The previously identified rabies isolates in different host species, such as badger, squirrel, donkey, cow, and goat across Jordan were related to antigenic variants, V1 and V6 [3]. The latter variant was identified for the first time on the border between Israel and Jordan [29]. Clades I, V, and VI appear to circulate in Jordan, where all isolates identified along the Israeli borders with Jordan. The RABV isolates of clade VI were identified in Syria as well, and closely related to Iranian isolates [3,29,30]. Meanwhile, clade I isolates circulate in Lebanon, Israel, and Jordan where identified along the Israeli borders with Jordan and Lebanon [3]. Indeed, the ME2 clade is closely associated with those from Turkey and Iran. The studies of whole genomes and the N gene sequences both confirm this. The previous Jordanian isolates belong to ME1a and 1b, and those are related to isolates from Saudi Arabia and Israel, which is expected since they are border countries with Jordan. It’s plausible that the new control efforts and regulations of wildlife across the border were beneficial. On the other hand, as most RABV isolates in this study were from the ME2 clade, closely to those from Turkey and Iran could be as a result of the continuous growth in the international travel movement in Jordan. Particularly, to and from Turkey in the last few years, where the number of Jordanian tourists raising from 162,866 in 2015 to around half million in 2019, besides that Turkey’s land borders with Iran [31]. Where travel is one of the risk factors for the emergence and spread of zoonotic diseases. Suggesting that rabies is a serious health concern throughout Jordan and its borders countries due to the travel and uncontrolled movement of stray dogs and wildlife animals (e.g. squirrels, jackals, foxes, wolves, monkeys, and stone marten) circulating the RABV across borders of neighbouring countries [3,4,32,33]. Further studies on RABV in Jordan and neighbouring countries such as Syria, Saudi Arabia, and Iraq are important to clarify the dynamics of rabies in the region.

The knowledge in disease epidemiology will help us to understand the spread of disease, evolution, and host restriction to identify effective control measures. These evolutionary and epidemiological analyses are effectively achieved by whole-genome sequencing studies of RABVs. The resulting molecular sequence combined with further analysis and bioinformatics tools will give insights into the processes of viral emergence in novel hosts and the adaptation of the latter, in addition to clarifying if RABVs are species-specific. As a result, improve our ability and response to develop effective monitoring programmes through rabies outbreaks, including vaccination and elimination enhancement. It is possible that the transmission of rabies virus is mediated by humans who bring infected animals with them while travelling [34]. In this case, education of the general public and policy makers would be important for controlling the spread of rabies. Also, in Islamic cultures in the Middle East, humans interaction with pets including dogs is limited compared to other cultures [35], thereby reducing the incidents of dog bites. Despite the growth in dog ownership and regulations in Jordan to regulate the licensing of dogs and limit the spread of stray and community-owned dogs according to Jordanian laws no. 138 and 154 published in the Official Gazette for the year 2016 [36], the problem still exists. Therefore, wildlife is likely to play an important role in the spread of rabies virus. Eliminating rabies virus in wildlife would ultimately reduce the threat to other animals and people.

In conclusion, rabies results in one of the highest death tolls of infectious diseases worldwide and is a global health concern, not only to human health but to animal health and food security [37]. Indeed, ME2 clade is closely associated with those from Turkey and Iran. The studies of whole genomes and the N gene sequences both confirm this. The previous Jordanian isolates belong to ME1a and 1b and those are related to isolates from Saudi Arabia and Israel. Therefore, there is a crucial need for strategies to control rabies, mainly through vaccinating dogs, the major reservoir of the disease. Also, as rabies has been eliminated in wild terrestrial mammals in Western Europe through oral vaccination programmes, the successful experience could be transferred to other low and middle income countries where rabies remains endemic. Finally, diagnostic tests including the FAT, RT-PCR and whole genome sequencing are reliable diagnostic tools for confirming rabies in animals and humans in Jordan. A considerable knowledge gap exists as a result of limited research collaboration in the field of transboundary diseases including rabies, which emphasizes the need for such collaborative networks to strengthen rabies surveillance in the Middle Eastern region.

## Supporting information

S1 Fig Map of Jordan and its border countries representing the locations of 7 rabies positive isolate samples (LN3, 2, 5, 9, 12, A, and B) across Jordan, which divided based on molecular classification into ME1a (green box) and ME2 (red boxes) clades. More information on the previously published RABV sequences from Jordan were displayed in the map of David et al., 2007 [3].
(TIF)

S2 Fig Representative microscopic images of Fluorescent antibody test (FAT). (A) The sample is negative for rabies and (LN1) (B) the sample is positive for rabies (LN5).
(TIF)

S3 Fig Percent identity and divergence generated by ClustalW (MegAlign, DNASTAR Version 15.0.0). (TIF)

S1 Table List of rabies suspect samples from Jordan analysed in this study. (DOCX)

S1 Appendix SNPs of core genome analysis. (XLSX)

## References

[1] Hampson K, Coudeville L, Lembo T, Sambo M, Kieffer A, Attlan M, et al. Estimating the global burden of endemic canine rabies. PLoS Negl Trop Dis. 2015;9(4):e0003709. 10.1371/journal.pntd.0003709 25881058 PMC4400070

[2] Sorrell EM, El Azhari M, Maswdeh N, Kornblet S, Standley CJ, Katz RL, et al. Mapping of Networks to Detect Priority Zoonoses in Jordan. Front Public Health. 2015;3:219. 10.3389/fpubh.2015.00219 26528460 PMC4600904

[3] David D, Hughes GJ, Yakobson BA, Davidson I, Un H, Aylan O, et al. Identification of novel canine rabies virus clades in the Middle East and North Africa. J Gen Virol. 2007;88(3):967–980. 10.1099/vir.0.82352-0 17325371

[4] Al-Qudah KM, Al-Rawashdeh OF, Abdul-Majeed M, Al-Ani FK. An epidemiological investigation of rabies in Jordan. Acta Vet. 1997;47(2/3):129–134.

[5] Su A. Jordan’s “Holy War on Dogs”—The Atlantic. Available online: https://www.theatlantic.com/international/archive/2017/11/jordans-holy-war-ondogs/546401. (Accessed on Feb 14. 2020).

[6] Faizee N, Hailat NQ, Ababneh MK, Hananeh WM, Muhaidat A. Pathological, Immunological and Molecular Diagnosis of Rabies in Clinically Suspected Animals of Different Species Using Four Detection in Jordan. TransboundEmerg Dis. 2012;59(2):154–164. 10.1111/j.1865-1682.2011.01255.x 22390575

[7] Crowcroft NS, Thampi N. The prevention and management of rabies. BMJ. 2015;350:g7827. 10.1136/bmj.g7827 25589091

[8] Leung AK, Davies HD, Hon KL. Rabies: Epidemiology, pathogenesis, and prophylaxis. AdvTher. 2007;24(6):1340–1347. 10.1007/BF02877781 18165217

[9] Hemachudha T, Ugolini G, Wacharapluesadee S, Sungkarat W, Shuangshoti S, Laothamatas J. Human rabies: Neuropathogenesis, diagnosis, and management. Lancet Neurol. 2013;12(5):498–513. 10.1016/S1474-4422(13)70038-3 23602163

[10] Mani CS, Murray DL. Rabies. Pediatr Rev. 2006;27(4):129–136. 10.1542/pir.27-4-129 16581953

[11] Singh R, Singh KP, Cherian S, Saminathan M, Kapoor S, Manjunatha GB, et al. Rabies–epidemiology, pathogenesis, public health concerns and advances in diagnosis and control: a comprehensive review. Vet Q. 2017;37(1):212–251. 10.1080/01652176.2017.1343516 28643547

[12] Nigg AJ, Walker PL. Overview, prevention, and treatment of rabies. Pharmacotherapy. 2009;29(10):1182–1195. 10.1592/phco.29.10.1182 19792992

[13] Nathwani D, McIntyre PG, White K, Shearer AJ, Reynolds N, Walker D, et al. Fatal Human Rabies Caused by European Bat Lyssavirus Type 2a Infection in Scotland. Clin Infect Dis. 2003;37(4):598–601. 10.1086/376641 12905146

[14] Matsumoto T, Ahmed K, Wimalaratne O, Yamada K, Nanayakkara S, Perera D, et al. Whole-genome analysis of a human rabies virus from Sri Lanka. Arch Virol. 2011;156(4):659–669. 10.1007/s00705-010-0905-8 21298456

[15] McElhinney LM, Marston DA, Freuling CM, Cragg W, Stankov S, Lalosević D, et al. Molecular diversity and evolutionary history of rabies virus strains circulating in the Balkans. J Gen Virol. 2011;92(9):2171–80. 10.1099/vir.0.032748-0 21632560

[16] Horton DL, McElhinney LM, Freuling CM, Marston DA, Banyard AC, Goharrriz H, et al. Complex epidemiology of a zoonotic disease in a culturally diverse region: phylogeography of rabies virus in the Middle East. PLoS Negl Trop Dis. 2015;9(3):e0003569. 10.1371/journal.pntd.0003569 25811659 PMC4374968

[17] Marston DA, Horton DL, Nunez J, Ellis RJ, Orton RJ, Johnson N, et al. Genetic analysis of a rabies virus host shift event reveals within-host viral dynamics in a new host. Virus Evol. 2017;3(2):vex038. 10.1093/ve/vex038 29255631 PMC5729694

[18] Dellicour S, Troupin C, Jahanbakhsh F, Salama A, Massoudi S, Moghaddam MK, et al. Using phylogeographic approaches to analyse the dispersal history, velocity and direction of viral lineages—Application to rabies virus spread in Iran. Molecular ecology. 2019;28(18):4335–50. 10.1111/mec.15222 31535448

[19] Troupin C, Dacheux L, Tanguy M, Sabeta C, Blanc H, Bouchier C, et al. Large-scale phylogenomic analysis reveals the complex evolutionary history of rabies virus in multiple carnivore hosts. PLoS pathogens. 2016;12(12):e1006041. 10.1371/journal.ppat.1006041 27977811 PMC5158080

[20] OiE Terrestrial Manual. 2018. Chapter 3.1.17. Rabies (infection with rabies virus and other lyssaviruses). Available online: https://www.oie.int/standard-setting/terrestrial-manual/access-online/ (accessed August 14, 2020).

[21] Marston DA, Jennings DL, MacLaren NC, Dorey-Robinson D, Fooks AR, Banyard AC, et al. Pan-lyssavirus Real Time RT-PCR for Rabies Diagnosis. J Vis Exp.2019;(149):e59709. 10.3791/59709 31355796

[22] Li H, and Durbin R. Fast and accurate long-read alignment with Burrows-Wheeler transform. Bioinformatics.2010;26:589–95. 10.1093/bioinformatics/btp698 20080505 PMC2828108

[23] Li H, Handsaker B, Wysoker A, Fennell T, Ruan J, Homer N, et al. The sequence alignment/map format and SAMtools. Bioinformatics. 2009;25(16):2078–2079. 10.1093/bioinformatics/btp352 19505943 PMC2723002

[24] Stamatakis A. RAxML version 8: a tool for phylogenetic analysis and post-analysis of large phylogenies. Bioinformatics. 2014;30(9):1312–1313. 10.1093/bioinformatics/btu033 24451623 PMC3998144

[25] Tonkin-Hill G, Lees JA, Bentley SD, Frost S, Corander J. RhierBAPS: An R implementation of the population clustering algorithm hierBAPS. Wellcome Open Res. 2018;3:93. 10.12688/wellcomeopenres.14694.1 30345380 PMC6178908

[26] Letunic I, & Bork P. Interactive tree of life (iTOL) v3: an online tool for the display and annotation of phylogenetic and other trees. Nucleic Acids Res. 2016;44(W1):W242–W245. 10.1093/nar/gkw290 27095192 PMC4987883

[27] Marston DA, Jennings DL, MacLaren NC, Dorey-Robinson D, Frooks AR, Banyard AC, et al. Pan-lyssavirus Real Time RT-PCR for Rabies Diagnosis. J Vis Exp. 2019;(149):10.3791/59709. 10.3791/59709 31355796

[28] McElhinney LM, Marston DA, Brookes SM, Fooks AR. Effects of carcase decomposition on rabies virus infectivity and detection. J Virol Methods. 2014;207:110–3. 10.1016/j.jviromet.2014.06.024 25010791

[29] David D, Yakobson B, Smith JS, Stram Y. Molecular epidemiology of rabies virus isolates from Israel and other middle-and Near-Eastern countries. Journal of Clinical Microbiology. 2000;38:755–62. 10.1128/JCM.38.2.755-762.2000 10655381 PMC86197

[30] Kissi B, Tordo N, Bourhy H. Genetic polymorphism in the rabies virus nucleoprotein gene. Virology. 1995;209:526–37. 10.1006/viro.1995.1285 7778285

[31] Republic of Turkey Ministry of Culture and Tourism, Tourism, Tourism Statistics, BORDER STATISTICS. Available online: https://www.ktb.gov.tr/EN-249299/yearly-bulletins.html [Accessed March, 31 2021].

[32] Bizri AR, Azar A, Salam N, Mokhbat J. Human rabies in Lebanon: lessons for control. Epidemiology & Infection. 2000;125:175–9. 10.1017/s0950268899004306 11057974 PMC2869584

[33] Yakobson BA, David D, Aldomy F. (2004). Rabies in Israel and Jordan. In Historical Perspective of Rabies in Europe and the Mediterranean Basin, pp. 171–183. Edited by King AA, Fooks AR, Aubert M, Wandeler AI. Paris, Geneva: World Organization for Animal Health (OIE) in conjunction with the WHO.

[34] Vega S, Lorenzo-Rebenaque L, Marin C, Domingo R, Fariñas F. Tackling the Threat of Rabies Reintroduction in Europe. Frontiers in Veterinary Science. 2021;7:1196. 10.3389/fvets.2020.613712 33521085 PMC7843519

[35] Alshehabat M, Obaidat M, Hayajneh W. Seroprevalence of Brucella canis in dogs and at-risk humans in Jordan. Veterinární medicína. 2019;64(6):260–5. 10.17221/67/2018-VETMED

[36] Hashemite Kingdome of Jordan, Official Gazette, rule no. 138 and 145/2016. Available online: http://www.pm.gov.jo/newspaperSubjects/5430/5430.html [Accessed March, 25 2021].

[37] Fooks AR, Cliquet F, Finke S, Freuling C, Hemachudha T, Mani RS, et al. Rabies. Nat Rev Dis Primers. 2017;30(3):17091. 10.1038/nrdp.2017.91 29188797

