# Supplementary figures and images for: Whole-genome sequencing and phylogenetic analysis of rabies viruses from Jordan
Source: PLoS Negl Trop Dis. 2021 May 20;15(5):e0009431. doi: 10.1371/journal.pntd.0009431 (PMC8171950; doi:10.1371/journal.pntd.0009431)

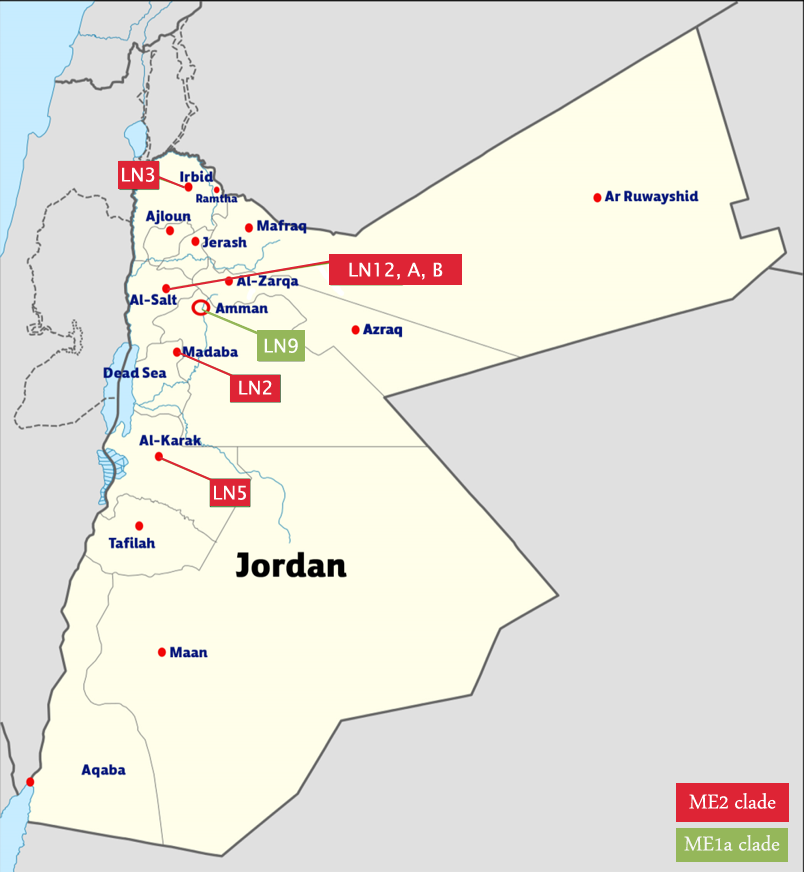

Supplement: S1 Fig — More information on the previously published RABV sequences from Jordan were displayed in the map of David et al., 2007 [3]. (TIF) [file pntd.0009431.s001.tif]

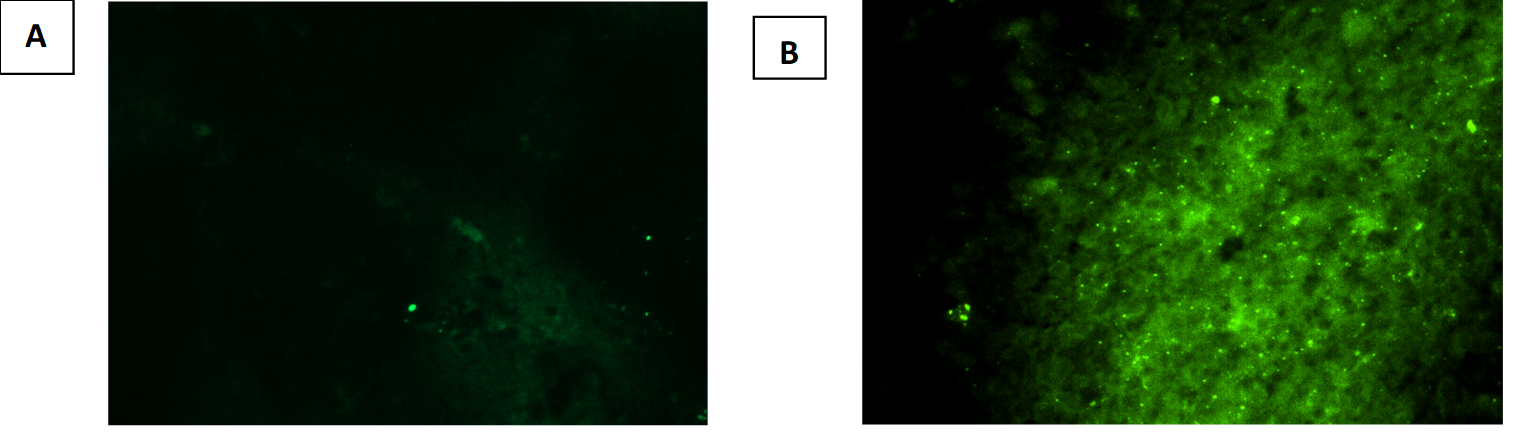

Supplement: S2 Fig — (A) The sample is negative for rabies and (LN1) (B) the sample is positive for rabies (LN5). (TIF) [file pntd.0009431.s002.tif]

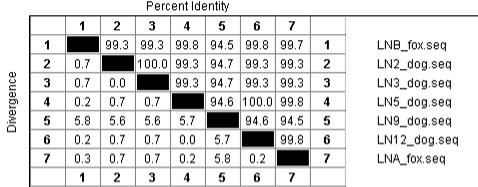

Supplement: S3 Fig — (TIF) [file pntd.0009431.s003.tif]
