# Supplementary material for: Whole-genome sequencing and phylogenetic analysis of rabies viruses from Jordan
Source: PLoS Negl Trop Dis. 2021 May 20;15(5):e0009431. doi: 10.1371/journal.pntd.0009431 (PMC8171950; doi:10.1371/journal.pntd.0009431)
Supplement: S1 Table — (DOCX) [file pntd.0009431.s004.docx]

| **S1 Table.** List of rabies suspect samples from Jordan analysed in this study. | | | | | |
| --- | --- | --- | --- | --- | --- |
| **Sample no.** | **Host** | **Town** | **Governorate** | **Date** | **FAT +PCR** |
| LN12 | Dog | DeirAlla | Balqa | 25/8/2019 | Positive |
| LN14 | Cow | Balqa | Balqa | 10/9/2019 | Negative |
| LN A | Fox | DeirAlla | Balqa | 21/12/2019 | Positive |
| LN B | Fox | DeirAlla | Balqa | 21/12/2019 | Positive |
| LN2 | Dog | German-Jordanian University | Madaba | 1/4/2019 | Positive |
| LN9 | Dog | Al Quwaysimah | Amman | 9/7/2019 | Positive |
| LN1 | Dog | Al-Shuna Al Shamalyah | Irbid | 25/3/2019 | Negative |
| LN3 | Dog | Al-Mazar Al-Shamali | Irbid | 24/4/2019 | Positive |
| LN7 | Cow | KufrAsad | Irbid | 23/6/2019 | Negative |
| LN15 | Sheep | Irbid | Irbid | 3/10/2019 | Negative |
| LN5 | Dog | Tafilah | Tafilah | 15/5/2019 | Positive |
| LN11 | Dog | Zarqa | Zarqa | 9/7/2019 | Negative |
